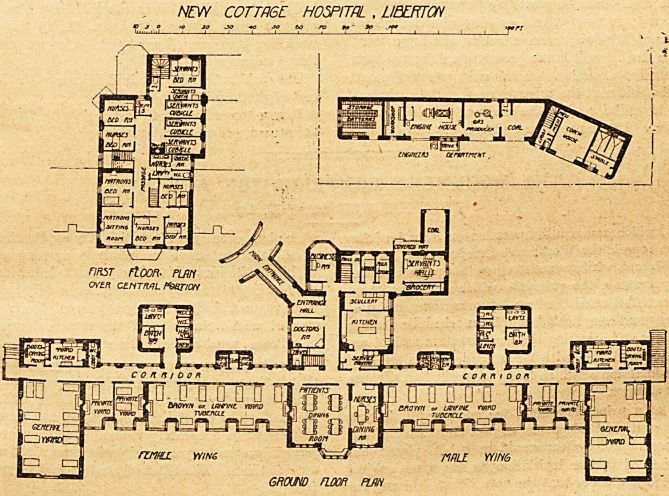# Edinburgh Hospital for Incurables at Liberton

**Published:** 1907-02-02

**Authors:** 


### EDINBURGH HOSPITAL FOR INCURABLES AT LIBERTON.

This is really an extension of the old hospital in Salisbury Place, Edinburgh, an institution which began as far back as 1875. It then contained only twenty-two beds, and was intended for the incurably sick from any part of Scotland, the only qualification being the need for constant medical attendance and skilled nursing. As might have been expected, the building was soon found to be inadequate to meet the calls made upon it; and in the year 1880 it was pulled down, giving place to a new hospital for fifty beds. In 1886, as further accommodation was required, two adjacent houses were added, which provided room for fourteen additional beds; but in a few years these houses were removed, and a large wing was built on the site. This addition afforded an opportunity of improving the sanitary arrangements, and the total accommodation was raised to 110 beds. In 1898 the west wing was added, and was devoted to the care of tubercle and cancer patients. The total number of beds was now 156.

For some years it had been evident that still further room was required; and the managers arrived at the satisfactory conclusion that it would not be desirable to enlarge the hospital in Salisbury Place, but that a separate building should be put up beyond the city boundaries. Of course, this meant more money; and it happened very opportunely that Miss Martha Brown, of Lanfine, left to the hospital the handsome amount of £20,000, of which £6,000 was to be expended in building, and the remaining £14,000 invested for purposes of endowment. It was wisely decided to combine the two schemes, by which a much better hospital could be obtained for less money than two separate institutions would cost.

It is clear, however, that the administration of the old hospital in Edinburgh and the new one at Liberton, however carefully managed, will require more funds, although the advantages to the patients will be much greater under the new system than under the old one. The managers say that a sum of £2,000 more than the present income will be needed, and they appeal to the public of Scotland to subscribe the additional sum. It can hardly be that their appeal will be in vain.

The new hospital is situate at Liberton, near Edinburgh. The site is four acres in extent, and it slopes towards the south. This falling away of the ground entails extensive under-building; but the advantage obtained in such cases is generally well worth the outlay. The building has a frontage of about 300 feet. It faces south, and all the wards have this aspect. The centre to the south is taken up by the patients' dining-room and by the nurses' mess-room, between which are large folding doors, whereby the two rooms may be thrown into one and used for entertainments. Next the dining-rooms is the tubercle ward for nine beds. In this the window embrasures are sufficiently large to admit of a bed being placed in them, so that the patient can have open windows on three aspects. There is, however, no verandah on which the beds could be placed in favourable weather. Next this ward are two private or single-bedded rooms, having the same window arrangement as the large ward. Next to these small wards is the general ward for ten beds. This ward has three windows on one side, one at the end, and one on the side next the tubercle ward. Both wings of the hospital are alike, and at the north of all these wards runs a corridor. In the partition wall between this corridor and the tubercle ward are several windows for cross-ventilation of the wards; and the door of the general ward also opens from the corridor. The tubercle ward would seem to have about 135 superficial feet and about 1,750 cubic feet of air space per bed, assuming a ceiling height of 13 feet; and the general ward a superficial space of 100 feet and a cubic space of 1,300 feet. These amounts strike us as being too low, especially when we look at the plan. The tubercle wards, for instance, have only one wall free to sun and air, as one end is blocked by the dining-room and the other by the private wards, although, as already said, the partition wall has openings, which will greatly increase the fresh air current. The general ward has the whole of one side and the whole of one end free; but half of the other side is blocked, and as the ward has only five windows, it follows that several beds have windows on one side only, while two beds have windows on neither side.

The great fault of the plan lies in the fact that the dining-rooms, tubercle wards, and general wards are continuous, instead of having interposed about eight or ten feet of corridor. The sanitary annexes are well placed and well designed, and they are carefully cut off from the main building.

North of the corridor and in the centre is the block containing the kitchen department, the office, the entrance hall, and the doctors' room. Over the whole extent of the centre are bedrooms and cubicles for the staff.

The wards are warmed by open fireplaces and by radiators. The floors and woodwork generally are of teak. The corridor is paved with blocks of marble. All the angles have been rounded off, and the mouldings, where there are any, are of the simplest form, so that there is not much fear of accumulation of dust.

The total number of beds in this, the Liberton Hospital, is 44, and, with the 156 in the Salisbury Place institution, makes a grand total of 200 beds. Truly a great and good work has been carried out by the managers during the last thirty years.

The architect of the new buildings was Mr. Peddie. The cost of the hospital is not stated.

It was formally opened last spring by Lord Dalkeith, in the presence of about 170 visitors.

## Figures and Tables

**Figure f1:**